# Relating to foetal persons: why women’s Voices come first and last, but not alone in Abortion debates

**DOI:** 10.1007/s11019-023-10144-0

**Published:** 2023-05-12

**Authors:** Stephen Milford

**Affiliations:** 1 Researcher at the Institute for Biomedical Ethics, Basel University, Basel, Switzerland; 2 Extra-ordinary Researcher, North-West University, Potchefstroom, South Africa

**Keywords:** Abortion, Pregnancy loss, Relational ontology, Relational personhood, Actual future principle, Pro-Live vs pro-choice

## Abstract

Abortion remains a controversial topic, with pro-life and pro-choice advocates clashing fiercely. However, public polling demonstrates that the vast majority of the Western public holds a middle position: being in favour of abortion but not in all circumstances nor at any time. The intuitions held by the majority seem to imply a contradiction: two early foetuses at the same point in development have different moral statuses. Providing coherent philosophical grounding for this intuition has proved challenging. Solutions given by philosophers such as Feinberg, Harman and Räsänen are complex and do not fully account for the lived experience of pregnancy loss. This article argues for a relational ontological construction of human personhood as the basis of foetal personhood. This approach takes seriously the literature of pregnancy loss and the lived experiences of pregnant persons. Focusing on the manner in which persons relate to early foetuses (especially pregnant persons), provides a coherent ground for distinct foetal value. Importantly, this approach is both simple and intuitive. Therefore, it can be more easily adopted by middle. To counter an implied equality of human relationality, the article argues for a clear hierarchy based on relational proximity that affirms pregnant persons? primary role in deciding the moral significance of foetal termination.

## Introduction

Ongoing public opinion polls – which have been stable for decades – indicate that the public is in favour of easily accessible abortions, but not at any time nor for any reason (Pew Research Centre 2022; YouGov 2022a; YouGov 2022b). To Langerak this is the ‘middle-of-the-road view’ (2014, 24) and is affirmed by the majority – at least in the West. On reflection, however, there appears to be a contradiction in this middle position. The public generally affirms widespread access to abortion and yet mourns a miscarriage, or is outraged at the ‘murder’ of an early foetus during a violent attack on a pregnant person (Collins 2015; Chalmers 2021). To Harman (1999) and Räsänen (2019) the contradiction lies in two widely held intuitions. The first is that we were all early foetuses, and it would have been wrong to harm us. The second is that nothing of moral significance takes place in elective abortion. If this middle-of-the-road position is to be respected, it needs good philosophical grounding.

Harman and Räsänen have attempted to provide this grounding by relying on complex philosophy involving Feinberg’s insistence on an actual present person as the object of a right, such as the right to life. Harman argues that such an actual person may apply in the case of a future *actual* person, what she calls the ‘actual future principle’ (1999). Were we to know that a foetus has no actual future (such as the case of the decision to terminate the pregnancy) then no person is harmed in abortion because there never *was to be* an actual person.^1^ Räsänen picks up Harman’s contention to argue for what he terms a ‘Schrödinger’s Fetus Model’ – foetuses are both actual and non-actual persons until a decision to continue or terminate the pregnancy is made (Räsänen 2019).

While philosophically brilliant in their construction, these models have two basic flaws. The first is their inability to account for the lived experience of those who have lost a wanted pregnancy. Harman insists that in such cases a pregnant person suffers under a ‘false belief’ and ‘they should also recognize that the death of the fetus should not be mourned’ (1999, 316). Yet the literature on pregnancy loss (often backed up by qualitative data) indicates that there is a strong belief among the public that something of moral significance has taken place in such cases. Second, their models lack the elegance of simplicity necessary to describe the widely held intuitions of the general public. Without this simplicity, it is unlikely that the public will easily adopt these models in defence of the middle ground against either ultra-conservative or ultra-liberal opponents.

Elsewhere we have dealt with the question of relational ontology and foetal status in depth. In this paper we pick up on an aspect alluded to in our past discussions: the practices that support a relational ontological approach to foetal personhood and in particular the key persons who enact these practices. For the uninitiated, the first two sub-sections of this paper provide a very brief summary of the challenges to foetal personhood and the relational ontological approach before considering the practical implications of this approach. Our paper here discusses some of the key practices that involved in the person-creating activity but are enacted by the community of persons. These practices directly impact the personal status of the foetus and ultimately its value within the community. Using a hierarchy of relational proximity, we argue that pregnant persons are the primary (albeit not sole) driver behind foetal value and that their experiences are to be taken as fundamental.

## The challenges of personhood in abortion debates

The question of foetal moral status is a question of personhood. As Kelsey (2009) rightly points out, it is persons that comprise a particular category of being whose membership are evaluated to have unqualified dignity resulting in certain rights. It is persons who have a right to life and therefore the *personal* status of the foetus is central to the ethical debates surrounding abortion (Manninen 2014). There are, however, a number of models by which personhood is describable – and consequently – ascribable to certain beings.

The first, and perhaps most widely referenced, is that of the substantive model. In this model, personhood is a substantive part of a being, central to their very existence. It is a characteristic, or feature, that cannot be removed without violating the unity and integrity of that being. There are many candidates for this characteristic, some have put forward humanities creativity, free-will, or the human mastery of the physical world (Cairns 1953; Hall 1986). In some cases, such as Van Rad, it is the human body with a specific genetic identity that universally sets human beings apart from other creatures and establishes their personhood (in Cairns 1953).

For thousands of years – at least in the traditional West – a concept of the human being as a rational animal has been perhaps the defining mark of anthropology. This is evidenced as far back as Aristotle (Aristotle, 1905*Pol.* I 2, 1523a7-18) and throughout Western Christian heritage from Augustine through Aquinas (Hill 1984; Grenz 2007), right up to Kant (Kant 1996; Roughley 2021). Recently the notion of rationality has been reinterpreted to focus on human self-reflection and consciousness. This, according to Olson (1997), is the ‘Standard View’ of personhood held by the majority of academic thinkers, and associates personhood with certain psychological features. That is to say; a person has certain psychological features associated with consciousness and any being who is not conscious, or at least will not be presently conscious, is not a person.

Naturally the implications of this view, when taken to their extremes, are unpalatable. As an example, one thinks of its ability to justify infanticide (Tooley 1985), a position that has recently received much attention in the recent back and forth debates between Räsänen and his critics (Räsänen 2016; Kaczor 2018 Räsänen 2018; Rodger et al. 2018; Rodger et al. 2018; Blackshaw and Rodger 2019). While understandably difficult to accept, it is hard to see how a substantive position does not lead to large portions of the human population being excluded from personhood and, consequently, having their value radically questioned (Milford 2018).

More than this, even those who do display characteristics associated with the Standard View do so only at certain points in their existence. The psychological features associated with self-reflection and consciousness are not always present throughout a human life. Not only is this the case of very young infants (who are hardly rational, conscious, self-reflective beings), but also for those who sleep, are in comas, or those who contract dementia and Alzheimer’s disease. So problematic is this aspect of the Standard View that to defend it Olson (1997) must distinguish between a personal identity and personhood, arguing that one has a personal identity throughout one’s life – as any other creature – and yet one is only a person during certain phases. In this way, personhood is used as a phasal sortal that designates members of the category of persons only at certain times (phases) during their biological careers (McMahan 2002, 2014). This strange re-interpretation of *personal identity* as a feature common to animals, yet distinct from personhood, is one reason to radically question the theory of animalism and the psychological view of personhood.

There is further challenge to the substantive model of personhood, and in particular the Standard View. This view describes all early foetuses as having the same ontological status. Either they are persons (through some metaphysical substantive reality not discussed here), non-persons (as they are not self-reflective), or merely potential persons. This indiscriminate ontological ascription is at the root of many abortion debates as opponents argue for the universal status of all early foetuses.

However, in practice, both in public polling and the lived experience of pregnant persons, early foetuses do not all have the same moral status, even though they share the same developmental state. Consider what we have alluded to in our introduction. The public broadly accepts abortion in certain circumstances and at certain times. This implies a diminished moral status for certain early foetuses. On the other hand, the public also broadly accepts that it is possible for an unborn child to be the victim of a crime, including homicide. This is evidenced in the Unborn Victims of Violence Act (2004 – abortion is specifically excluded). While few cases are actually prosecuted under such laws, there is general public outcry whenever a wanted pregnancy is lost as a result of a violent crime (Collins 2015; Chalmers 2021).

Furthermore, there appears a contradiction when a pro-abortion activist mourns the loss of a wanted pregnancy. Even Harman, who argues that no person has been harmed in this case, acknowledges the serious trauma but ultimately concludes that the pregnant person was mistaken to think that their foetus had moral value. To Harman, the pregnant person may mourn the loss of their own desires, and express upset that they must start again, but they cannot mourn the loss of a being that was the legitimate object of love, or that would have been wrong to have harmed (Harman 1999).

Harman’s position, however, is disputed in the literature on pregnancy loss (Parsons 2010; Lindemann 2013; Wright 2018; Chambers 2020). Here authors argue that the lived experience of those who have lost (through natural or unnatural means) a wanted pregnancy is radically distinct from those who have lost (actively or passively) their unwanted foetuses. The experience of such persons is that their unborn foetuses were of moral significance, the legitimate objects of love, and the kinds of things that it would have been wrong to have harmed. To these people, their early foetuses were personal.

## Relational persons, not conscious great apes

A possible solution to the conundrum as to why different foetuses at the same stage of development have different moral statuses may lie in the recent shift in some sections of anthropology towards a more social or relational approach to personhood (Shults 2003). Within this framework, personhood does not denote a substantive quality of a being, but a particular relationship certain beings have with each other, namely: personal relationships. Here, personhood is eccentrically conceived (McFadyen 1990; Kelsey 2009; Milford 2019); that is, external to the entity in question. For the Yale professor David Kelsey (2009), it is as we are personally related to by other persons (who are themselves so conceived), that we become persons. Thus, *personal identity* denotes a particular sense of identity: that aspect of our identity that describes the types of relationships we have with other personal identities. To Kelsey, it is personal relations that come before persons. We are personalised by these relationships and as such are created as persons (Chambers 2020). Consequently, persons are beings who are ‘called into personhood’ by other persons (cf. McFadyen 1990; Lindemann 2013).

Of course, there are some concerns about a relational ontological approach to human personhood. In particular, it is possible that such an eccentric conception might lead to an unhealthy relativity rooted in a never-ending regression whereby persons, create persons who create persons. We have discussed relational anthropology in depth elsewhere, including the objections of unending relativity (Milford 2018, 2019, 2020). We leave those discussions for now and take it at face value that personhood speaks primarily to the types of relationships certain beings have with each other, rather than a substantive ontological reality.

Engelhardt argues that the mother-child relationship epitomises this aspect of personhood. A very young infant displays very few of the characteristics traditionally associated with personhood. It is not rational, self-reflective or – as far as we can tell – self-conscious. Yet the mother treats the early infant as if it were a person. She calls it by name, comforts it as if it were a person, and even speaks to it as if one would speak to another person. This is in spite of the infant not comprehending the meaning of her words. As the mother acts in these ways – in personal ways – she personalises her child and therefore, within the mother-child relationship, the child has the status of a person (Engelhardt 1973).

Engelhardt’s model need not start at birth. Indeed, there is ample evidence in the literature on pregnancy loss to demonstrate that this model is in place long before birth. Space prohibits an extensive discussion of this point here, but there is value in drawing attention to one or two examples of the personalising relationship between a pregnant person and their foetus.

## Naming, declaring, and placing early foetal persons

To begin with we can note the terms used by pregnant persons. In wanted pregnancies, it is often considered cold and ‘impersonal’ to use the term *embryo*, or *foetus*. This is considered clinical and may even be offensive outside medical contexts. Instead, pregnant persons often refer to their foetuses as ‘baby.’ Many may go so far as to use substitute proper names such as Bean, Bump, Bug, Bun etc. These terms are important. Unlike clinical terms such as *embryo; foetus;* or *infant* – which are used to categorise entities into similar groups – proper names are used to distinguish beings from each other. They denote personal identity, an identity that is personal to that particular entity in question. By using these terms as individual placeholders for future proper names, pregnant persons understand their foetuses as being personal beings.

Second, we can note the testimony of the pregnant person to the personal status of their foetus. In wanted pregnancies, the pregnant person often testifies about the impending arrival of a new person to their community of persons. Billions of dollars a year are spent on these testimonies which take individualised forms. This includes ultrasonic pictures of the foetus, personalised announcements on social media, gender reveal parties, and regular updates as to how the foetus and pregnancy is progressing. Not only does this demonstrate how excited the pregnant person is about their foetus, but one of the aims of these testimonies is to prepare the community of persons for the arrival of a new member. The pregnant person hopes that their particular community (friends and family) will become exited as well and welcome the new foetal person into their community.

Third, we can note the objects involved in the pregnancy. In many cases, those engaged in a wanted pregnancy will make use of personalised objects. For example, they will begin to create a space for the infant by decorating the nursery, or by choosing clothes and soft toys. They will receive personalised gifts at a baby shower (that takes place before the child is born) which may include objects of a certain colour or may even be personally inscribed with the foetus’ future name. These rituals – decorating rooms, throwing baby showers etc. – are all with the expressed purpose of personalising both the child and the pregnancy experience so as to deepen the personal bond between mother and child.

These are just three ways in which persons engaged in wanted pregnancies treat their foetus as if it were a person. By giving it a name, announcing it to the community, and preparing a space in the home, the pregnant person personalises their foetus and in these ways engages in the ‘person-creation project’ (Chambers 2020). We could mention many other personalising activities: dreaming of their future child, stroking their abdomen, staring at ultrasound pictures, attending pre-natal classes with other persons engaged in the person-creating project etc. While none of these activities on its own creates a person, their collective effort has the result of personalising the infant and creating a person.

## Women’s voices – first and last

What is significant about this construction of personhood is that it is persons who create persons. In particular, it is the pregnant person who has the primary role in the person-creating project. It is the pregnant person who enacts many of the practices noted above: names the foetus, announces its impending arrival, dreams of its future, prepares a place for it in the home and the community. It is, therefore, the pregnant person who is primarily responsible for whether or not it becomes a person.

This is an important point. There are many foetuses who are not personalised by the persons carrying them. The reasons for this are numerous. It may be that the pregnant person does not know they are pregnant. It may be that there is no time before the event of a natural miscarriage. In some cases, for whatever reason, the pregnant person actively chooses not to engage in the person-creating project. For example, where their mental health is compromised, or where the pregnancy is the result of a traumatic experience (we think of cases of rape, or arising from abusive situations). In these cases the pregnant person may actively choose not to relate to their foetus in personalising ways.

In many of the cases mentioned above, termination may result (natural or chosen). In this case the termination was not that of a person, not even a potential person. This is because the pregnant person had not begun to personalise their foetus, nor did they have the intention of doing so. Consequently, no person would have ever resulted from this pregnancy and therefore nothing of moral significance has taken place in its termination. On this particular point we side with Fienberg (1981), Harman (1999), and Räsänen (2019).

The consequence of our position, therefore, is to place the majority of responsibility for the moral or a-moral nature of an active termination with the pregnant person. Using a construction based on relational ontology, it is the pregnant person who ultimately decides if a termination had moral significance or not. In cases where they had not personally related to the foetus, nor had any intention of ever doing so, then it is perfectly understandable that they may feel no moral guilt regarding the termination. Indeed, it is coherent. On the other hand, as is the experience of many persons who have experienced pregnancy loss, in cases where they had begun to personalise their foetus, or had the firm intention of doing so, the loss of this foetus (both natural or unnatural – as in the case of an unwanted but unavoidable termination) has moral significance. It is coherent that the pregnant person may mourn the loss of a person (their child). Here we diverge from authors such as Harman and Räsänen who claim that the pregnant person is simply ‘mistaken’ to mourn the loss of a wanted pregnancy on the basis of the loss of a person. Therefore, within a relational ontological construction, it is the pregnant person who has the first and last say about the nature of the termination of their own pregnancy.

## Women’s voices – not alone

Not negating what is said above, it is important to point out that while the pregnant person has the primary say, they do not speak alone. Recall that relational ontology speaks primarily to personhood as a member of a community of persons. That is to say, persons (plural) create persons. We noted above that to be a person is to belong to a particular category of beings, beings who relate to each other in personal ways. This category is, by definition, communal. It is the community of persons who create persons.

Relational ontology has arisen over the last few decades in part as a protest against the radical individualisation that has plagued the West since the Enlightenment (Lukes 1973; Soares 2018). For many centuries the West has been obsessed with the autonomy of the individual often to the exclusion of the community. Recently the majority world has criticised this philosophy and offered an alternate view of humanity. For example, within Ubuntu philosophy – epitomised in Mbiti’s adage: ‘The individual can only say: “I am because we are; and since we are, therefore I am” (Mbiti 1969, 106) – there is an age old saying: ‘it takes a village to raise a child.’ Within such a construction, to ignore the wider community of persons in a discussion about what happens to a person (potential or actual), is to ignore the very foundation of personhood itself.

Recall the three examples of personalising activities enacted by pregnant persons we mentioned above. These three enactments take place within the community. First, to name the foetus using a personal pronoun such as *Bean* is partly to declare to the community that they too are to utilise this name as a substitute pronoun until the foetus is born and given its proper name. In many cases, the pregnant person’s partner is active in choosing this substitute pronoun. Once it has been chosen, and is being used by the pregnant pair, their community is often encouraged to also use personalising terms when talking about the foetus. Rarely at the dinner table will the future grandparents talk about the zygote, embryo, foetus or even infant. They will normally speak of baby, child and often make use of the couples preferred substitute pronouns. This goes not only for grandparents, but for aunts, uncles, friends, midwives, and the wider community.

In like fashion, as the pregnant person (and their partner) testify to the community of persons of the impending arrival of another person, they engage the community actively. Pictures are shared, messages passed on and in due course it is likely that the community will begin to testify to each other of the impending arrival. Interestingly, the desire of the community to testify often goes beyond the wishes of the pregnant person, and it is not uncommon for an over eager grandparent to share news that a pregnant person would have liked to have shared themselves, resulting in some minor tentions.

Third, in preparing a space for the foetus among the community, the pregnant person often involves members of their community of persons. For example, baby showers are opportunities for other people to provide personalised objects to help prepare the home for the foetus. The pregnant person’s partner may help pick out the crib, paint the nursery, or install the new car seat. The community becomes engaged in helping prepare for the arrival of a new member of their community and as they do so they further contribute to the personalising activities of the pregnant person.

Therefore, while it is true that the pregnant person is central to the person-creating project, they enact these activities in the context of a community who is often actively engaged in this same project. Consequently, to argue that one need only hear the voice of the pregnant person within the context of debates about abortion is to fail to understand the very nature of personhood. The community of persons has a vested interest in the person-creating project and should not be left out.

## A clear hierarchy

That the community is involved in the person-creating project presents a significant challenge that must be addressed. It is possible that the community attempts to personalise the foetus against the wishes of the pregnant person. This can be the case, for example, when the biological father has a divergent opinion on the pregnancy than the pregnant person themselves. He can, for example, believe that the foetus is a person and therefore attempt to use personalising pronouns, community announcements, and even attempt to make a space for the foetus in the community even though the pregnant person is not so inclined. Such personalising can, unfortunately, go beyond merely the pregnant person’s partner, and in some cases entire communities feel that their personalising actions outweigh the feelings and opinions of the pregnant person themselves. Sadly, this has been the case in many contexts in which communities have implemented blanket bans on abortions. In these cases entire communities act against the wishes of the pregnant person. This has forced pregnant people into very difficult (even life-threatening) situations – in some cases even when no foetal person is viable.

These communal approaches should not be taken as positive expressions of communalism in protest of Western individualism. Quite the contrary, they can be forms of communal oppressions of individual rights, resulting in appalling abuses. Therefore, it is important that the role of the community in the personalising of early foetuses is placed in an appropriate perspective. While we are here advocating that the community has a role to play in the person-creating project and are thereby instrumental in the personalizing of early foetuses, we are not advocating that this role supersedes that of the pregnant person’s primary responsibility. Quite the contrary, we propose a clear hierarchy of personalising relationships based on relational proximity.

The first person on this hierarchical ladder is the pregnant person themselves. They are unquestionably closest in proximity to their own foetus. This includes physically/biologically but also relationally. Not only do they spend the most time with their early foetus out of any member of the community, but on the whole also the most energy in personalising the foetus. It is often the pregnant person who engages most in the types of personalising activities we have noted above: speaks of their foetus (naming it and declaring it to the community of persons), preparing space for it, spending time dreaming about it, going shopping for personal items etc. This close physical, relational, and personalising proximity is arguably the primary driving force behind the person-creating activity. Therefore, it is only natural to argue that the pregnant person’s voice is both first and last in the personal status of their early foetus.

Second on the hierarchical ladder is the pregnant person’s partner. The partner is often both physically and relationally closer than any other community member. It is often they who need to carry some of the added burden the pregnancy brings on the family, they who next speak most of their future infant (both to the pregnant person and the community). The partner often accompanies the pregnant person to medical appointments, shopping trips, and assists in preparing the home. It is, therefore, very understandable that they would want to be involved in decisions of terminations and will keenly mourn the loss of their foetus. Their close proximity to the early foetus means that they too have a vested interest in the person-creating activity, and while decisions over termination ultimately rest with the pregnant person, their voice should be considered as a close second.

Beyond the partner, the wider community plays a decreasingly important role. Close family (siblings, parents etc.) are often both physically and relationally closer in proximity than friends and neighbours. Nevertheless, all these still have vested invests in the person-creating activity undertaken by a pregnant person and should not be completely ignored. Members of the community who are not physically and relationally close to a pregnant person may well have interests in what happens to early foetuses, but their interests are far removed. Their voices can (and should) be heard, but their influence over the personal nature of an individual early foetus wanes with an extending relational proximity.

## Conclusion

The middle ground has long been trodden on by the extremes in abortion debates (Manninen 2014). The loud minority calling for either a total ban or a free-for-all have silenced the majority who hold an intuitive position displaying a *prima facia* contradiction. Yet, the middle-ground is neither coldly rational nor overly emotional. Their position, that some foetuses have moral value and others not, is coherent when viewed through a relational ontological lens. Understanding personhood as primarily a relational category has a long history in philosophy (Shults 2003) and is intuitive. Therefore, reframing abortion debates away from the standard view of foetal personhood toward a more social view has two key advantages.

First, as we have shown, it provides a coherent reason why some early foetuses are the kinds of things it would be wrong to harm, and at the same time nothing of moral significance takes place in the termination of an unwanted pregnancy. Second, it is intuitively simple. That personal relationships are the foundations of human dignity and value is not only rationally intelligible but is the daily lived experience of all human beings. That a mother mourns a miscarriage is not simply a mistake, it is both intellectually logical and emotionally understandable. Consequently, a relational ontological approach to debates surrounding abortion provide the ordinary person with a simple rational defence of their position that is intuitive and rooted in their lived experience.

However, in order to avoid the tyranny of both communalism (that attempts to ban all terminations to protect potential persons) and individualism (that disregards the opinion of the community of persons), a relational approach needs to promote a clear hierarchy based on relational proximity. Every member of the personal community has a voice, but not all voices are of equal weight. Driven by the force of personal relationality in personalising early foetuses, those who are relationally closest to the foetus have a naturally more significant say on the personal status of early foetuses. Ultimately this entails that the pregnant person’s voice is primary. It is first and last even if it is not alone.

## Footnotes

1 For more on the distinctive intrinsic value of a potentiality or actuality see (Burgess 2010; Feinberg 1981, 2014; Manninen 2014; McMahan 2014). Here we note merely that an aborted foetus was at no time even a potential person.

## References

[CR1] Aristotle (1905). Aristotle’s politics.

[CR2] Blackshaw, Bruce P., and Daniel Rodger. 2019. Why a right to life rules out infanticide: A final reply to Räsänen. *Bioethics* 33. Wiley-Blackwell: 965–967. 10.1111/bioe.12646 31389040

[CR3] Burgess JA (2010). Potential and foetal value: Journal of Applied Philosophy. Journal of Applied Philosophy.

[CR4] Cairns David (1953). The image of God in Man.

[CR5] Chalmers, Robbie. 2021. Perthshire mum launches campaign to protect unborn victims of domestic violence. *Daily Record*. July 23.

[CR6] Chambers K, Lindsey (2020). It’s complicated: what our attitudes toward pregnancy, abortion, and Miscarriage tell us about the Moral Status of early fetuses. Canadian Journal of Philosophy.

[CR7] Collins, Kat. 2015. “Too hard to convict people of harming unborn babies.” *BBC News*, April 17, sec. Newsbeat.

[CR8] Engelhardt H, Tristram (1973). Beginnings of Personhood: philosophical considerations. Perkins Journal.

[CR9] Feinberg Joel (1981). The rights of animals and unborn generations. Responsibilities to future generations.

[CR10] Feinberg Joel, Lizza John P (2014). The Paradoxes of Potentiality. Potentiality: metaphysical and bioethical dimensions.

[CR11] Grenz, Stanley J. 2007. *The Social God and the relational self: a trinitarian theology of the Imago Dei*. 1st ed. Westminster John Knox.

[CR12] Hall Douglas John (1986). Imaging God: Dominion as Stewardship.

[CR13] Harman, Elizabeth. 1999. Creation Ethics: The Moral Status of Early Fetuses and the Ethics of Abortion. *Philosophy* & *Public Affairs* 28. Princeton University Press: 310–324. JSTOR Journals. 10.1111/j.1088-4963.1999.00310.x 11657937

[CR14] Hill Edmund (1984). Being human: a biblical perspective.

[CR15] Kaczor Christopher (2018). A Dubious Defense of “After-Birth Abortion”: a reply to Räsänen. Bioethics.

[CR16] Kant Immanuel, Gregor Mary J (1996). Groundwork of the Metaphysics of Morals. Practical philosophy.

[CR17] Kelsey David H (2009). Eccentric existence: a theological Anthropology.

[CR18] Langerak Edward, Lizza John P (2014). Abortion: listening to the Middle. Potentiality: metaphysical and bioethical dimensions.

[CR19] Lindemann Hilde (2013). Holding and letting go: the Social Practice of Personal Identities.

[CR20] Lukes Steven (1973). Individualism.

[CR21] Manninen Bertha Alvarez, Lizza John P (2014). Revisiting the argument from fetal potential. Potentiality: metaphysical and bioethical dimensions.

[CR22] Mbiti John S (1969). African Religions & Philosophy.

[CR23] McFadyen Alistair Iain (1990). The call to Personhood: a christian theory of the individual in Social Relationships.

[CR24] McMahan Jeff (2002). The Ethics of Killing: problems at the margins of life.

[CR25] McMahan Jeff, Lizza John P (2014). Potentiality. Potentiality: metaphysical and bioethical dimensions.

[CR26] Milford, Stephen R. 2018. Substantive or relational? The Counterfeit choice in the Imago Dei Debate. *McMaster Journal of Theology and Ministry* 20.

[CR27] Milford Stephen R (2019). Eccentricity in Anthropology: David H. Kelsey’s Anthropological Formula as a way out of the substantive-relational Imago Dei Debate.

[CR28] Milford Stephen R (2020). The Problem with Sandra: addressing the unfortunate Consequences of Relational Ontological Personhood. Religion and Theology.

[CR29] Olson, Eric T. 1997. Was I Ever a Fetus? *Philosophy and Phenomenological Research* 57. Buffalo, N.Y: International Phenomenological Society: 95–110.

[CR30] Parsons Kate (2010). Feminist reflections on Miscarriage, in light of abortion. International Journal of Feminist Approaches to Bioethics.

[CR31] Pew Research Centre. 2022. Public Opinion on Abortion. *Pew Research Center’s Religion* & *Public Life Project*.

[CR32] Räsänen Joona (2016). Pro-life arguments against Infanticide and why they are not convincing. Bioethics.

[CR33] Räsänen Joona (2018). Why pro-life arguments still are not convincing: a reply to my critics. Bioethics.

[CR34] Räsänen Joona (2019). Schrödinger’s Fetus. Medicine, Health Care and Philosophy: a european Journal.

[CR35] Rodger Daniel, Blackshaw Bruce P, Wilcox Clinton (2018). Why arguments against Infanticide remain Convincing: a reply to Räsänen. Bioethics.

[CR36] Roughley, Neil. 2021. Human Nature. In *The Stanford Encyclopedia of Philosophy*, ed. Edward N. Zalta, Spring 2021. Metaphysics Research Lab, Stanford University.

[CR37] Shults F, LeRon (2003). Reforming Theological Anthropology: after the philosophical turn to Relationality.

[CR38] Soares Conceição (2018). The philosophy of individualism: a critical perspective. International Journal of Philosophy and Social Values.

[CR39] Tooley, Michael. 1985. *Abortion and infanticide*. Clarendon.

[CR40] Wright Lenore J (2018). Relationality and life: phenomenological reflections on Miscarriage. International Journal of Feminist Approaches to Bioethics.

[CR41] YouGov. 2022a. Should women have the right to an abortion? *YouGov*.

[CR42] YouGov. 2022b. Should the legal time limit to have an abortion change? *YouGov*.

